# To Bag or Not to Bag? How AudioMoth-Based Passive Acoustic Monitoring Is Impacted by Protective Coverings

**DOI:** 10.3390/s23167287

**Published:** 2023-08-20

**Authors:** Patrick E. Osborne, Tatiana Alvares-Sanches, Paul R. White

**Affiliations:** 1 School of Geography and Environmental Science, Faculty of Environmental and Life Sciences, University of Southampton, Southampton SO17 1BJ, UK; tatiana.alvares-sanches@newcastle.ac.uk; 2 School of Computing, Urban Sciences Building, Newcastle University, Newcastle upon Tyne NE4 5TG, UK; 3 Institute of Sound and Vibration Research, Faculty of Engineering and Physical Sciences, University of Southampton, Southampton SO17 1BJ, UK; p.r.white@soton.ac.uk

**Keywords:** acoustic indices, acoustic metrics, AudioMoth, calibration, frequency response, passive acoustic monitoring

## Abstract

Bare board AudioMoth recorders offer a low-cost, open-source solution to passive acoustic monitoring (PAM) but need protecting in an enclosure. We were concerned that the choice of enclosure may alter the spectral characteristics of recordings. We focus on polythene bags as the simplest enclosure and assess how their use affects acoustic metrics. Using an anechoic chamber, a series of pure sinusoidal tones from 100 Hz to 20 kHz were recorded on 10 AudioMoth devices and a calibrated Class 1 sound level meter. The recordings were made on bare board AudioMoth devices, as well as after covering them with different bags. Linear phase finite impulse response filters were designed to replicate the frequency response functions between the incident pressure wave and the recorded signals. We applied these filters to ~1000 sound recordings to assess the effects of the AudioMoth and the bags on 19 acoustic metrics. While bare board AudioMoth showed very consistent spectral responses with accentuation in the higher frequencies, bag enclosures led to significant and erratic attenuation inconsistent between frequencies. Few acoustic metrics were insensitive to this uncertainty, rendering index comparisons unreliable. Biases due to enclosures on PAM devices may need to be considered when choosing appropriate acoustic indices for ecological studies. Archived recordings without adequate metadata may potentially produce biased acoustic index values and should be treated cautiously.

## 1. Introduction

In recent years, advances in technology have led to a surge in studies employing passive acoustic monitoring (PAM) as a surveillance technique in environmental and ecological contexts [1,2,3] A reduction in the cost of hardware now means it is often possible to deploy, simultaneously, multiple devices in a single study, increasing the area covered and the amount of data collected. One such low-cost device for PAM is AudioMoth [4]. The AudioMoth couples a low-power microcontroller with an analogue microelectromechanical systems (MEMS) microphone [4,5]. It is capable of recording and storing sounds over a wide frequency range, from anthropogenic noise of around 1 kHz, through audible wildlife at 4 kHz and up to 192 kHz for wildlife using the ultrasonic range [6]. AudioMoth recorders have successfully been used in a wide variety of applications, from remotely monitoring wild mammals [7,8,9], to identifying bird and frog species from spectral signatures using a convolutional neural network [10] and even assessing exposure to urban noise on building façades [11].

AudioMoth devices are sold as bare electronic boards (around USD 97 in 2023 for the latest version from https://www.labmaker.org/products/audiomoth-v1-2-0 (accessed on 6 August 2023)) and, for most applications, need protecting from environmental factors in an enclosure. Recently, a proprietary injection-moulded polycarbonate case has become available, featuring a Porelle AV51D acoustic vent for the microphone (https://github.com/OpenAcousticDevices/Application-Notes/blob/master/An_Injection_Moulded_Case_for_AudioMoth/An_Injection_Moulded_Case_for_AudioMoth.pdf (accessed on 6 August 2023)). However, this adds around USD 40 to the price (https://www.labmaker.org/products/audiomoth-ipx7-case (accessed on 6 August 2023)), and alternative home-made solutions have included simple food containers, enclosures made from DIY components and 3D-printed cases (see https://www.openacousticdevices.info/support/enclosures/summary-of-enclosures-to-date (accessed on 19 August 2023) for a useful summary). The simplest, however, are zip-closure polythene bags that are both widely available and cheap. Global availability is important, as lost or damaged bags may be replaced locally, even in remote situations. Plastic bags have been illustrated as suitable enclosures by the AudioMoth developers [4,5] and on users’ blogs, although sometimes with reported condensation problems and ingress of water (https://www.openacousticdevices.info/support/enclosures/be-carefull-with-zip-lock (accessed on 19 August 2023)). In our experience, plastic bags can give suitable protection from rain, although, in windy conditions, the repeated rubbing of the plastic on the edges of the AudioMoth electronic board can cause splitting and leakage.

Very little information appears to have been published on how any of these housings affect the acoustic performance of the devices, the exception being [12] as part of a wider study. This is potentially of concern, for example, given the care and attention that has gone into the development of microphone windshields over many decades. If blocking the wind and rain is as simple as using a plastic bag, why are wind mufflers so expensive? Our study was motivated by the concern that the choice of housing (and therefore microphone covering) may potentially alter the spectral response of the device. Depending on the research project, this may not matter because, for example, small shifts in spectral response have minor effects on how a recording sounds, and species identification aurally should still be possible. However, as the range of projects using AudioMoth and other PAM devices diversifies, housings could alter frequency responses in ways that compromise comparisons of data from either within a single study or among different studies.

In this paper, we focus on plastic bags as the simplest housing solution for AudioMoth recorders, although the principles (but not the details) apply to other recording devices and their enclosures. Using an anechoic chamber, we performed a series of experiments to assess how plastic bags affect the values of 19 acoustic metrics and ecoacoustic indices calculated from AudioMoth recordings. At least some of these feature in many studies employing PAM [13,14,15,16,17,18]. Simple acoustic metrics, such as mean frequency, can obviously be affected by shifts in frequency response, while measures such as RMS (average amount of energy per unit time) may be reduced by anything obstructing the microphone. Ecoacoustic indices are routinely employed in ecological studies yet are potentially more problematic because they are often based on the relative amounts of energy in different frequency bands. Changes in the frequency responses of PAM devices due to the housings could render comparisons of such indices across devices and sites unreliable, potentially misleading the otherwise laudable goal of developing conservation action from audio information [19].

This paper addresses three research questions that potentially influence the ways in which PAM devices should be used in ecological studies, illustrated through an examination of the AudioMoth:

(i) Do AudioMoth recorders require calibration to capture the true frequency composition of the source signal?
(ii) Is the need for calibration affected by the housing?
(iii) What is the impact of the lack of calibration on calculated acoustic metrics and ecoacoustic indices?

Our goal is not to criticise current practice but to point the way to expanded use of PAM devices through an understanding of limitations imposed by frequency responses and housings.

## 2. Materials and Methods

Ten AudioMoth recorders (version 1.1.0, obtained from different sources) were programmed to record at a sampling rate of 48 kHz with the gain set to medium. Each recorder was tested as a bare board, and then again with three experimental bag configurations (Table 1), each with only a single layer of plastic covering the microphone.

The bags were fitted in a standard way by just one researcher, who had previously carried out the same process more than 100 times, to maintain consistency. Experiment B2 in Table 1 (repeat bagging of the same device) refers to incomplete data recorded incidentally to the main trial when testing for appropriate sound levels for the experiment. We did not initially intend to analyse these data, but they reveal important clues to consistency, a key point we refer to later.

The devices were held at a 1.42 m height by a microphone clamp, directly facing a loudspeaker (Genelec 8020C, Iisalmi, Finland) placed 1.98 m away at the same height. A series of pure sinusoidal tones in deci-decades from 100 Hz to 19.95 kHz were generated using MATLAB (R2021a, Mathworks, Massachusetts, UK), rendered via a DAQ System (NI USB-6341; National Instruments, Theale, UK) and played through the speaker and recorded on the AudioMoth devices. The same source signals were also recorded on a calibrated Brüel & Kjær Class 1 sound level meter (SLM, Lübeck, Germany, type 2250) placed in exactly the same location as the AudioMoth relative to the speaker. All the experiments were conducted in the large anechoic chamber at the University of Southampton with minimal noise intrusion.

Combining the SLM data and the AudioMoth recordings of the broadband noise, the frequency response function (FRF) between the incident pressure wave and the recorded signal at the AudioMoth was calculated. A linear phase finite impulse response (FIR) filter with 513 taps was designed with the same FRF as that computed for each of the AudioMoth configurations. Applying this filter to the data replicates the effect of the AudioMoth and any bag on that data and provides a convenient way to investigate how sounds are actually recorded.

To assess how the AudioMoth and the bags affect derived acoustic indices, we used our archive of over 250,000 sound clips recorded across the city of Southampton during 2020. Although these were collected using AudioMoth, they are simply being used here as example sound sources with analyses only considering how these are affected by the filters simulating the FRF of the AudioMoth with and without bags. Used in this way, it should not matter what the origins of the recordings were. To ensure we had a good range of signal characteristics among our sound clips, we took stratified random samples from the archive using three indicative metrics (RMS, ADI and ACI—Table 2), which had already been processed from the recordings without correction, to yield raw acoustic indices. To achieve this, the entire archive was stratified into 10 quantile groups based on each metric, and an approximately equal number of samples was drawn at random from each quantile to select 333 sound clips per metric. The stratified samples for each metric were then combined and duplicates removed, yielding a set of 998 unique sound recordings with a wide range of acoustic characteristics.

We assessed how our filters (and therefore the AudioMoth recordings made with and without bags) affect 19 representative acoustic metrics and ecoacoustic indices, grouped into four families (Table 2; colour-coded for convenience). Most of these metrics are conveniently coded in R [20], although Table 2 gives the original citations, where known. To assess impact, we plotted the unfiltered metric (representing the true source signal) against the filtered metrics (AudioMoth/bag effects). If there were no effects of the experimental treatment on the indices, we would expect the relationships to follow the line of equality (i.e., a 1:1 line). Deviations from this line were assessed using Willmott’s *md*, borrowed from hydrology [21]:

(1) $$md=1-\frac{\sum_{i=1}^{N}|S_{i\;}-A_{i\;}|}{\sum_{i=1}^{N}|A_{i}-\bar{S}\;\left|+\right|{\;S}_{i\;}-\bar{S}|}$$

where *S_i_* is the acoustic index value from source signal *i*, and *A_i_* is the corresponding value from the AudioMoth recording. A value of *md* = 1 indicates perfect agreement, whereas *md* = 0 indicates no agreement.

We also assessed consistency in the calculated acoustic metric values between tests of the same individual sound clip on different AudioMoth devices (with or without bags) using a form of the coefficient of variation:

(2) $$CV=100|\frac{Mean[Within\;clip\;SD\;of\;\left(A_{i\;}-S_{i\;}\right)]}{Grand\;mean\;of\;(A_{i\;}-S_{i\;})}|$$

where *S_i_* is the index value from source signal *i*, and *A_i_* is the corresponding value from the AudioMoth. Values of *CV* = 0 indicate perfect consistency, ranging to plus infinity for no agreement.

## 3. Results

### 3.1. Calibration

**Figure 1:**
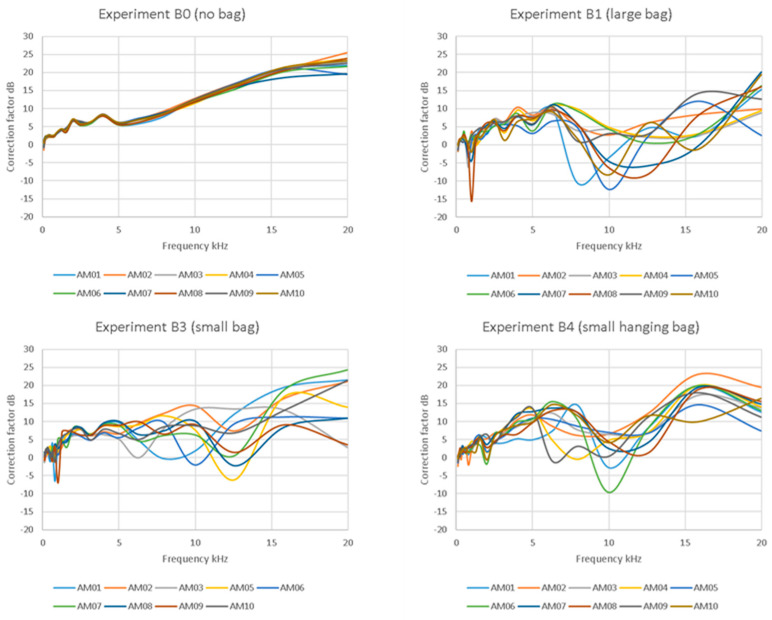
FRFs of AudioMoth with and without the bag treatments in Table 1. The correction factor is the amount that needs to be subtracted from the recording on the AudioMoth to recover the source signal. The AudioMoth FRFs are consistently coded, AM01 to AM10, to allow comparisons between graphs. Note that AM04 has been omitted from experiment B3 due to a recording error.

The FRFs of the bare board AudioMoth (B0 in Table 1) were remarkably consistent below about 13 kHz, after which small variations were observed between devices (Figure 1). However, the FRFs were not flat, showing substantial accentuation of higher frequencies relative to the lower frequencies and with almost 25 dB variation in the averaged response across the frequency band (100 Hz–20 kHz).

**Figure 2:**
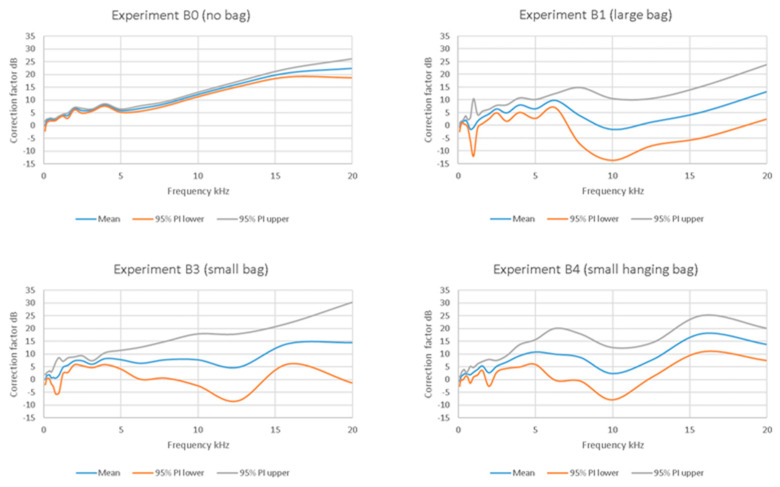
Mean and 95% prediction intervals (PI) for the FRFs for the four experimental treatments. The prediction intervals (PI) show the limits within which 95% of all responses are likely to lie.

Placing the AudioMoths in bags had a marked effect on the spectral responses that were, apparently, often highly inconsistent (Figure 1). In all the bag configurations, there was a tendency for frequency responses to show greater repeatability at low frequencies (below 2–3 kHz), particularly for experiment B4, but unpredictable behaviour above 5 kHz, leading to wide 95% prediction intervals (Figure 2). All the bags reduced the response at a high frequency relative to that for the bare AudioMoth (e.g., see mean trends in Figure 2), but in an inconsistent manner.

**Figure 3:**
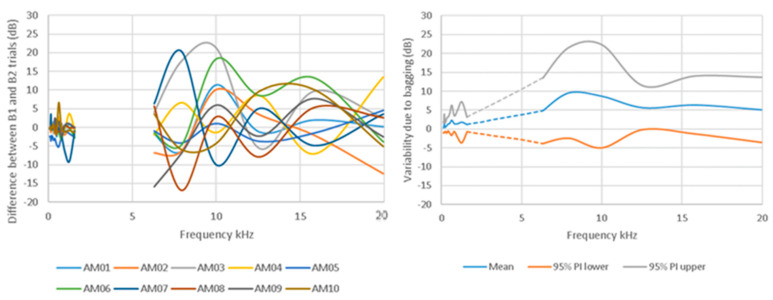
The left-hand figure shows the difference between dB levels recorded at each frequency tested for experiments B1 and B2, i.e., repeat bagging of each AudioMoth. Ideally, all values should be zero. As there is no logical ordering between trials B1 and B2, the right-hand figure shows the mean and 95% prediction intervals (PI) for the absolute difference between repeat bagging. The dotted lines have been added for visualisation only and cover frequencies for which data were lacking.

As the bare board AudioMoth showed very consistent FRFs, while the bagged devices did not, the bags themselves (or the action of bagging the AudioMoth devices) must be responsible. Indeed, analysis of the incomplete data from the pre-trial (experiment B2 in Table 1, initially intended only to set audio levels) showed a large variation between repeat bagging of the same AudioMoth (Figure 3). In other words, putting the AudioMoth in the bag introduced variations in the FRF, and repeating the operation on the same device, in the same way, did not result in the same outcome. On average, across all frequencies and AudioMoth devices tested, using the large bag (B1 and B2) added variations in the response of ±2.9 dB. However, low frequencies were less affected than higher frequencies, and variability peaked at around 8 kHz, with a mean uncertainty of ~9.6 dB with 95% PI of −2.4 to 21.6 dB (Figure 3).

### 3.2. Bag Effects on Metrics

**Figure 4:**
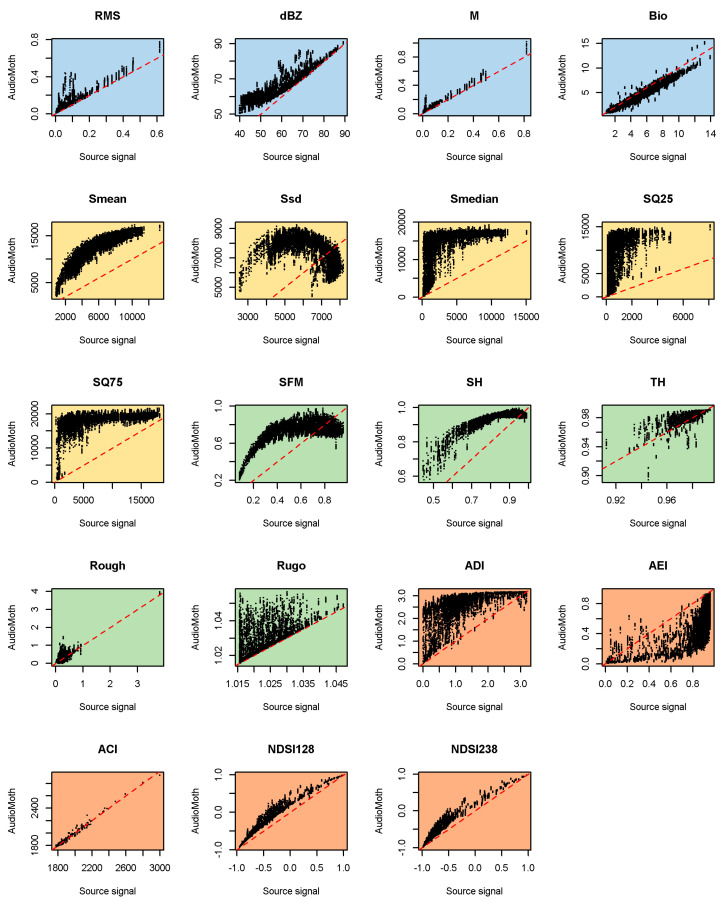
Comparison of acoustic metric values derived from 998 source signals and as recorded on 10 AudioMoths with no bag. The dashed line is the line of agreement. The metrics are described and colour-coded in Table 2.

**Figure 5:**
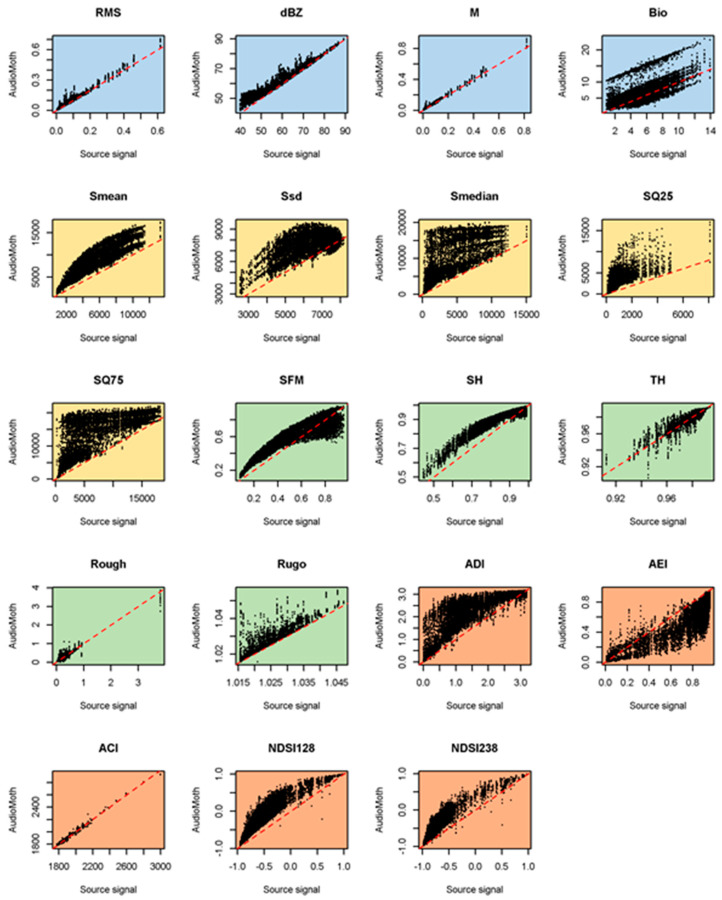
Comparison of acoustic metric values derived from 998 source signals and as recorded on 10 AudioMoths with a B1 bag. The dashed line is the line of agreement. The metrics are described and colour-coded in Table 2.

**Figure 6:**
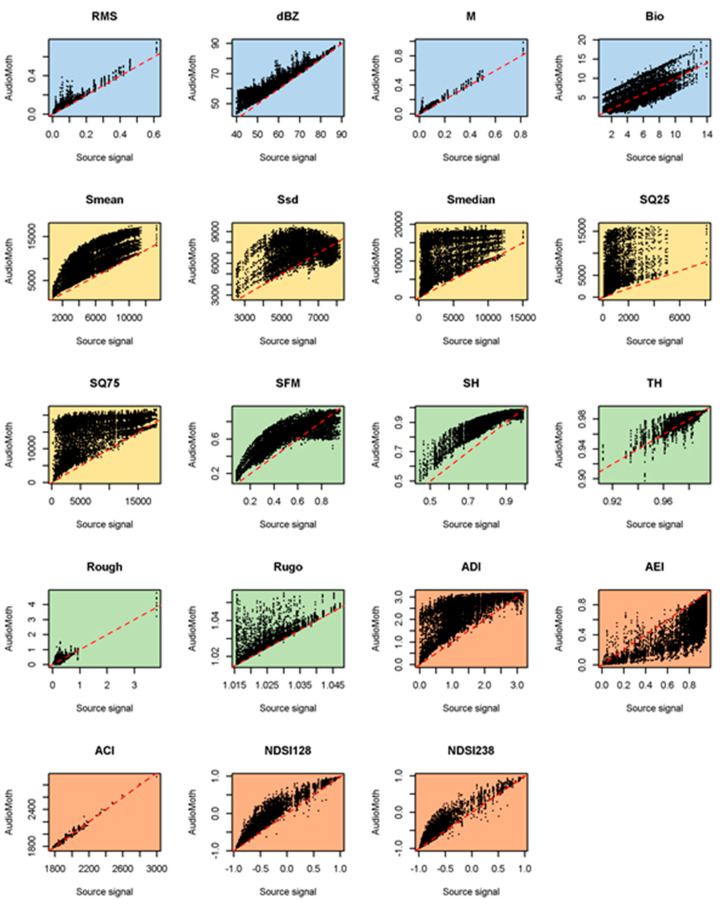
Comparison of acoustic metric values derived from 998 source signals and as recorded on 9 AudioMoths (one failed during the experiment) with a B3 bag. The dashed line is the line of agreement. The metrics are described and colour-coded in Table 2.

**Figure 7:**
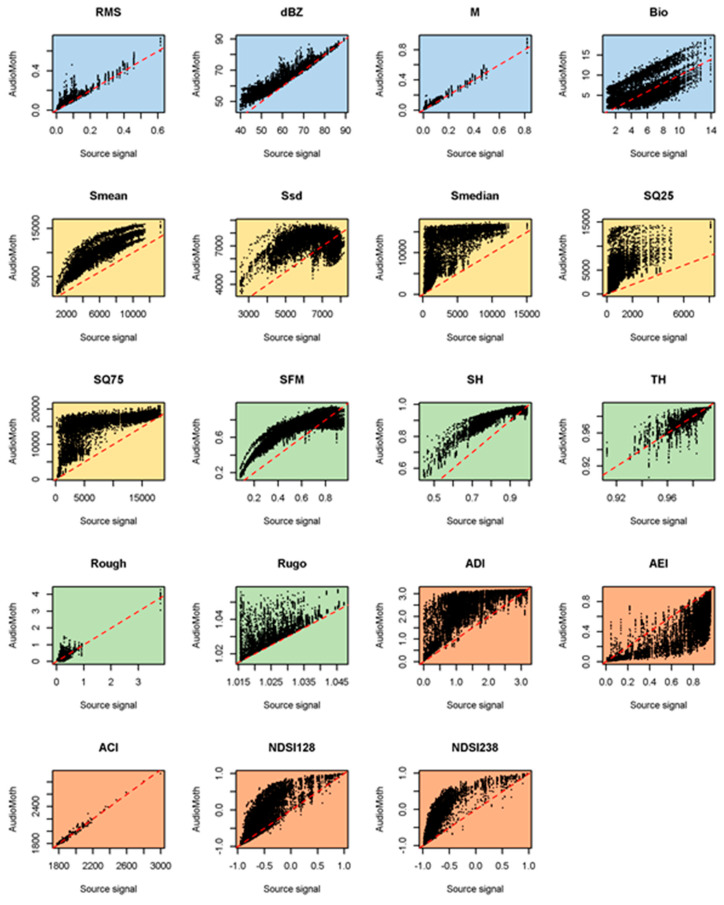
Comparison of acoustic metric values derived from 998 source signals and as recorded on 10 AudioMoths with a B4 bag. The dashed line is the line of agreement. The metrics are described and colour-coded in Table 2.

When we calculated the acoustic metrics (Table 2) from the AudioMoth recordings, we found strong differences compared with the metrics calculated from the source signals (Figure 4, Figure 5, Figure 6 and Figure 7; data in [32]), even on bare board AudioMoths (Figure 4).

As the bare board AudioMoth tended to add high-frequency emphasis (accentuating the higher frequencies relative to the lower ones; Figure 1), all the metrics related to the dominant frequency (Smean, Ssd, Smedian, SQ25 and SQ75—colour-coded yellow in Table 2 and Figure 4) were badly affected (i.e., strongly deviating from a 1:1 line). A visual inspection of Figure 4 shows that among the ecoacoustic indices (coloured orange) recorded on the bare board AudioMoth, ADI and AEI were the worst affected, followed by the two NDSI metrics and then ACI, the least affected. The bare board AudioMoth had a tendency to overestimate metrics based on the time domain (Figure 4; coloured blue), although Bio was underestimated. The metrics based on geometric properties (Figure 4; green) were highly skewed on the bare board AudioMoth recordings.

The metrics calculated from the AudioMoth devices in bags showed modified responses that depended on how they were bagged and the metric used (Figure 5, Figure 6 and Figure 7). Among the time-domain metrics (blue), M was relatively little affected by the bags, whereas the response of Bio was split into multiple parallel lines. None of the set-ups tested faithfully captured dBZ as would be needed for noise assessments, the best being the B1 bag, especially above 75 dB. This is likely to reflect that noise sources contributing most to the sound field were low-frequency, where the bag was the least influential.

Ecoacoustic indices (orange in Figure 5, Figure 6 and Figure 7) tended to deviate more from a 1:1 line when calculated from bagged than bare board AudioMoth (e.g., NDSI), although ACI remained consistent and true to the source signal throughout. As with the metric M, indices based on statistical properties (yellow in Figure 5, Figure 6 and Figure 7) often split into multiple parallel curves in the bagged devices. Visually, the metrics based on geometric properties (green) sometimes showed a closer fit to a 1:1 line when recorded in a bagged AudioMoth (e.g., SFM), presumably because the housing reduced the high-frequency content.

This visual impression of the effects of the bags was confirmed by goodness-of-fit tests (Willmott’s *md* statistic) applied to the data (Table 3). Despite the rather chaotic nature of the frequency responses in Figure 1, AudioMoths in B1 bags actually showed the closest acoustic metric matches to the source signal, even compared with the bare boards. This was true for all the metrics except ACI (which performed equally well across bag treatments), NDSI128, NDSI238 and Bio.

According to Willmott’s *md*, the acoustic metrics most robust against the effects of the method of deployment were ACI (all configurations), M (with B1), RMS (with B1) and TH (with B1) (Table 3). Note, however, that the latter still showed a wide spread of values (Figure 5).

The results in Table 3 are somewhat counter-intuitive, because of the consistency of the frequency responses among the bare AudioMoth (Figure 1), and can be explored further by looking at the variation in the calculated acoustic metric values between tests of the same sound clip on different devices (Table 4). The index in Table 4 captures the spread on the *y*-axis for any given value on the *x*-axis that arises due to variations between AudioMoth/bag combinations (i.e., it removes the effects of different sound sources leading to coincident acoustic index values). Looked at in this way, the bare AudioMoth clearly performed best across all the metrics except for the metric Rough, which had a peculiarly clumped range of values (Figure 4, Figure 5, Figure 6 and Figure 7). Given that AudioMoth cannot be deployed in the field without protection from the weather, it is instructive to examine the second-best values for each metric (italics in Table 4). In this case, the B4 bag performed best for 15 out of 19 metrics. Note, however, that second-best (italics) values were nearly always substantially worse than the best values (bold) because of the way bags affect the frequency responses (Table 4).

The relative contributions of the source signal, bag, and individual device were investigated by estimating their effect sizes within linear models (Table 5) using the R package effectsize [33]. The results are approximate only due to the assumptions around linearity and interacting effects, but provide an indication of the relative contributions. In all cases except for Ssd (which showed a highly curvilinear relationship in Figure 4), the primary contribution to variance in the AudioMoth recorded signals came from the source signal itself, as might be expected (Table 5). Confirming previous results, the acoustic metrics with the highest effect size (partial eta squared) associated with the source signal (and therefore the most robust across deployments) were ACI, M, RMS and dBZ in decreasing order. The bag effects were most severe for Smean, dBZ, NDSI238 and then, jointly, SFM and SH.

## 4. Discussion

### 4.1. Frequency Response of the AudioMoth

The FRFs of the bare board AudioMoth were very consistent but accentuated higher frequencies. Previous work [12] has reported only small variations in the spectral responses between new AudioMoth, but our devices had already been deployed in the field several times yet still gave consistent results. However, our data do not show relatively flat frequency responses in contrast to [12]. When we listened to recordings made on our devices, we found no difficulty in identifying bird species, despite the high-frequency emphasis, suggesting that human aural identification is not impacted. This is where the purpose of the study needs to be clear from the start: if aural identification is all that is required, the frequency shifts present little problem. Additionally, if bare board AudioMoth are being used only for comparative assessments of acoustic indices, the consistency between devices suggests the correct rank order of sites (for example, in acoustic diversity) would be obtained. However, the absolute values of many acoustic indices are heavily impacted by frequency response, and they should not be compared across studies employing different forms of PAM recorders or housings.

### 4.2. Effect of the Bag on this Frequency Response

Of course, AudioMoth recorders can rarely be deployed in the field without protection from the weather, and we found that the addition of a protective plastic bag led to unpredictable frequency responses that were inconsistent between bag changes and modes of deployment. This was despite all the bag changes being made by just one researcher who had done the process many times before and in a repeatable manner. For example, the AudioMoth was always placed in one corner of the B1 bag with a single layer of plastic over the microphone, any excess being folded behind the battery pack. This finding, based on more extensive testing, differs from the conclusion in a previous study that plastic bags had relatively little effect on spectral response [12], although they did note attenuation due to the bags above 10 kHz.

Given the consistency in the performance of bare board AudioMoth devices, the frequency spectrum shifts for enclosed AudioMoth in Figure 1 arise solely from the bags and do not reflect differences between circuit boards. Comparing our experimental treatments (Table 1), we could hypothesize that both bag thickness and folding affect frequency response. Thicker plastic could potentially attenuate signals more, although it is difficult to understand how this happens at the frequencies studied because the wavelengths were all greater than 14 mm, far longer than the bag thickness. Additionally, our B1/B2 bags were only 15% thicker than the B3/B4 bags. A comparison of our B3 and B4 experiments suggests that folding the excess bag behind the device might have an effect, perhaps because signals are reflected from the folds, even though there is a battery pack in-between in all cases. If reflections matter this much, it also suggests that how and where the AudioMoth is attached to a structure (branch, wall, etc.) has an impact. We carried out our experiments in an anechoic chamber with minimal reflections off surfaces, but, in the real world, signals will arrive at an AudioMoth from multiple directions and angles (see also [12]).

Unfortunately, precise analytical prediction of the impact of the bag on the acoustics of the recorder is not feasible. There are several factors which confound such analysis, two of which are the fact that the membrane (i.e., the bag) and microphone are in close proximity, so near-field effects are to be expected; and, secondly, that the edges of the membrane are not tensioned in a controlled manner, so the boundary conditions are ill-defined. However, we can observe some trends in the data which conform with general predictions for simplistic models. Specifically, the bag is seen to introduce low levels of attenuation at low frequencies (below 5 kHz), which is consistent with predictions based on the mass law [34]. This is with the exception of some narrow bands at low frequency, e.g., close to 1 kHz, where higher attenuations are observed, and this we attribute to local resonant behaviour. At higher frequencies, more complex behaviour is evident, which is presumably the result of various phenomena, including standing waves on the membrane and within the structure of the AudioMoth itself. From a practical point of view, our tests showed that the hanging bag with no folds had the least impact on the acoustic indices after the bare board AudioMoth (B4 in Table 4), and this might be the best arrangement for field use. Ironically, because the responses of AudioMoths in bags are (on average) flattened, as high frequencies are being attenuated, the results from the bagged data are possibly closer to the raw signal (again, on average), but with greater variation. In the absence of further data, our advice is to avoid using bags if possible, and always to be as consistent as possible in the deployment method if the study is one that requires spectral information from PAM devices in protective housings, i.e., using the same housings in exactly the same way throughout and attaching the recorders to the same types of support in the same way across the study area. Even with these precautions, expect frequency shifts from the source signals in unpredictable ways.

### 4.3. Choice of Acoustic Metrics

When audio signals are altered by the frequency response of a device, and then further modified by the housing used (e.g., bag, container), the spectral characteristics of the recording can differ markedly from those of the source signal. This has a knock-on effect on the values of a wide range of acoustic metrics that may be calculated. However, the effects are not uniform, and our empirical tests showed that some metrics are more robust against frequency changes. On the bare board AudioMoth, M, ACI, NDSI and Bio showed reasonable resilience, although the effects are not always linear (Figure 4). When bags were used, M and ACI remained among the least affected metrics. The robustness of ACI can be explained because it looks at the energy within a frequency bin and sums temporal differences in those energies. That sum is then normalised by the total energy and summed across bands. This means that if a gain is applied to a band, ACI will remain unchanged. The effect of the FRFs can be approximated as applying different gains to different bands, which therefore leaves ACI unchanged. RMS was also fairly robust in some configurations but requires calibration [35] to have more than a comparative meaning. Indices such as ADI, AEI, Bio and NDSI cannot generally be recommended from recordings made on AudioMoths in bags unless correction factors are employed. Again, it is crucial to consider the potential limitations in any field deployment carefully and to choose acoustic indices depending on those constraints.

The effects reported here are in addition to the biases that can arise in the use of acoustic indices due to community composition and the intrusion of extraneous sounds [36,37,38,39]. Often, no single index is sufficient, and several will be needed to characterise an area [13]. Furthermore, there are limitations in the effectiveness of acoustic indices to quantify biodiversity, and caution is needed when using them as surrogates for biodiversity metrics [40]. Our analysis has intentionally compared AudioMoth recordings with source signal characteristics across a wide spectrum of index values, but there are obvious variations in how well the data fit a 1:1 line, depending on the index value. For example, the middle of the range for RMS with our B1 bag (Figure 5) appeared to fit tolerably well, suggesting that the effects we report may not limit studies narrowly focused on particular index values. This is an extension of the idea that the choice of index should depend on the acoustic properties of the source signals expected, such as who or what is making the sound [38].

## 5. Conclusions

In this experimental study, we have shown that AudioMoth recorders show a consistent frequency response, at least in our sample of 10 devices. They do, however, accentuate high frequencies and therefore require calibration to capture the true frequency composition of source signals. Unfortunately, the calibration needed is affected by plastic bag housings, often in unpredictable ways that appear to vary between fittings of the same bag in the same way. Both the accentuation of high frequencies by the bare board AudioMoth and the use of housings affect the values of most acoustic metrics and ecoacoustic indices calculated from sound recordings. These limitations must be borne in mind when planning field studies; some projects will be affected, whereas others will not. It is also vital that metadata accompanies any archived recordings in order to limit misleading conclusions that could arise from researchers re-analysing old data without knowledge of the way the recording devices were deployed.

We are strong supporters of PAM, and devices such as the AudioMoth have greatly advanced the ability of researchers to capture environmental sounds across space, time and the frequency spectrum. Our goal in writing this paper has not been to criticise these revolutionary developments, but rather to help guide the expansion of their use into other fields. With careful spectral calibration (and, for some studies, calibration of sound levels), low-cost sensors such as the AudioMoth can be successfully used across many applications. As new enclosures come onto the market and the ability to fit external microphones is explored (https://github.com/OpenAcousticDevices/Application-Notes/blob/master/Using_AudioMoth_with_External_Electret_Condenser_Microphones/Using_AudioMoth_with_External_Electret_Condenser_Microphones.pdf (accessed on 19 August 2023)), the range of these applications is set to grow and add to the field of environmental acoustics. We have explored just one way of protecting AudioMoth in the field, and the newer, hard casings may give different results [12]. Importantly, if it is the flexibility of the bag that causes unpredictable variations in spectral response, the harder casings may perform more consistently, and this would be advantageous. Although we have not formally investigated the acoustic performance of the new proprietary housing (https://www.labmaker.org/products/audiomoth-ipx7-case (accessed on 6 August 2023)), preliminary trials suggest strong attenuation (up to 10 dB) below about 1.3 kHz and amplification above 2 kHz, but this clearly needs additional work. There is some evidence, however, that recordings made on AudioMoth mounted in the new housing are sufficiently faithful to the original to be classified correctly by automated recognition software [41], which bodes well for future studies.

## Figures and Tables

**Table 1:** Experimental deployments used for the ten AudioMoth recorders. The onboard microphone was at the top left in all deployments.

Experiment	Deployment of AudioMoth	Typical Set-Up
B0	Bare board (i.e., no bag). Held by clamp underneath.	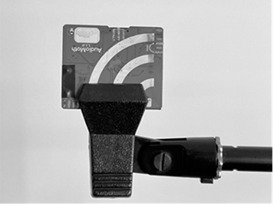
B1 B2	Large (235 × 285 mm), slightly thicker (47 gm^−2^) bag with surplus folded behind the device, kept in place with cable tie. Held by clamp underneath. B2 repeats B1 after re-bagging.	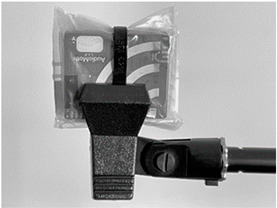
B3	Small (95 × 150 mm), slightly thinner (41 gm^−2^) bag with surplus folded behind the device. Held by clamp underneath.	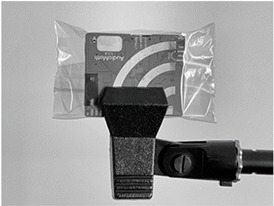
B4	Small (95 × 150 mm), slightly thinner (41 gm^−2^) bag with no folds, suspended from a clamp.	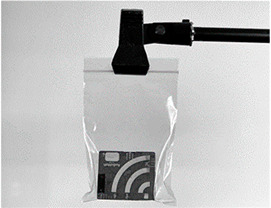

**Table 2:** Acoustic indices and their definitions as applied here. The shadings represent the different families of metrics. The measures highlighted in blue are based on time-domain properties (although computations may be done in the frequency domain). Those in yellow are based on statistical properties of the spectrum; in green are metrics based on geometric properties of the spectrum; and in orange are quantities based on manipulations of the energies in one or more frequency bands.

Metric	Description
RMS	**Root mean square** is the square root of the average amount of energy in the clip per unit time. For use in ecological research, see [22].
dBZ	**Unweighted sound level**, calculated across the entire frequency spectrum, i.e., 20 log10(RMS) + 94
M	**Median** of the signal envelope, scaled by bit depth [23]
Bio	**Bioacoustic index** energy in the signal filtered between 1 and 8 kHz [24,25]
Smean	The **mean** frequency of the frequency spectrum [20]
Ssd	The **standard deviation** of the mean frequency of the spectrum [20]
Smedian	The **median** frequency of the spectrum [23]
SQ25	The frequency at the **first quartile** of the frequency spectrum [20]
SQ75	The frequency at the **third quartile** of the frequency spectrum [20]
SFM	**Spectral flatness measure** is the ratio between the geometric mean and arithmetic mean among frequency bins of the frequency spectrum [26]
SH	**Shannon evenness among the frequency bins** of the frequency spectrum [27]
TH	The temporal amplitude index, assessing the **Shannon evenness of the amplitude envelope** [27]
Rough	**Roughness** captures the curvature of the frequency spectrum curve and is the integrated squared second derivative of the spectrum [20]
Rugo	**Rugosity** is similar to roughness but instead based on the first derivative [20]
ADI	**Acoustic diversity index** of the energy content in frequency bands between 0 and 10 kHz above a threshold, here −40 dB [28,29]
AEI	**Acoustic evenness index** uses the Gini coefficient to assess the equity of the energy content distribution among frequency bands, defined as in ADI [29]
ACI	**Acoustic complexity index**, measuring the complexity of the signal but giving most importance to sounds that are modulated in amplitude rather than consistent [30]
NDSI128	**Normalized difference soundscape index** contrasting the energy in the 1 to 2 kHz frequency bin (anthropophony) against that in the 2 to 8 kHz frequency bins (biophony) [31]
NDSI238	**Normalized difference soundscape index** contrasting the energy in the 2 to 3 kHz frequency bin (anthropophony) against that in the 3 to 8 kHz frequency bins (biophony) [31]

**Table 3:** Willmott’s *md* values for how close the fits in Figure 4, Figure 5, Figure 6 and Figure 7 are to a 1:1 relationship. Values in bold are the highest (best) in each row. The metrics are described in Table 2.

Metric	Bare	B1 Bag	B3 Bag	B4 Bag
RMS	0.75	**0.90**	0.83	0.83
dBZ	0.57	**0.83**	0.72	0.71
M	0.73	**0.90**	0.82	0.82
Bio	**0.81**	0.59	0.67	0.52
Smean	0.28	**0.50**	0.44	0.40
Ssd	0.07	**0.41**	0.35	0.26
Smedian	0.31	**0.58**	0.51	0.48
SQ25	0.47	**0.57**	0.53	0.50
SQ75	0.15	**0.52**	0.42	0.34
SFM	0.28	**0.69**	0.57	0.51
SH	0.25	**0.57**	0.46	0.40
TH	0.74	**0.88**	0.84	0.82
Rough	0.77	**0.84**	0.83	0.80
Rugo	0.72	**0.85**	0.83	0.78
ADI	0.34	**0.57**	0.50	0.45
AEI	0.44	**0.67**	0.59	0.55
ACI	**0.94**	**0.94**	**0.94**	**0.94**
NDSI128	**0.74**	0.60	0.70	0.67
NDSI238	0.78	0.68	**0.79**	0.60

**Table 4:** Consistency (CV) of acoustic metric values between different methods of AudioMoth deployment. Values in bold are the lowest in each row, indicating the least variation between device/bag set-ups. Values in italics are the second best. The acoustic metrics are described in Table 2.

Metric	Bare	B1 Bag	B3 Bag	B4 Bag
RMS	**15.8**	45.2	43.7	*31.3*
dBZ	**9.6**	35.4	37.8	*21.4*
M	**16.4**	49.4	47.1	*32.0*
Bio	**21.6**	*180.0*	326.5	299.1
Smean	**9.1**	42.8	55.3	*22.9*
Ssd	**15.6**	53.3	88.8	*39.9*
Smedian	**9.9**	51.6	60.3	*27.7*
SQ25	**13.5**	41.1	66.6	*39.4*
SQ75	**6.4**	43.8	51.9	*19.5*
SFM	**10.4**	86.8	58.5	*32.9*
SH	**5.4**	21.5	27.4	*13.8*
TH	**19.6**	112.5	112.6	*74.6*
Rough	*338.2*	**172.9**	476.5	872.2
Rugo	**33.2**	*37.3*	77.4	52.4
ADI	**6.3**	22.9	28.7	*14.9*
AEI	**8.7**	34.4	38.4	*22.8*
ACI	**1.9**	13.5	7.9	*6.1*
NDSI128	**7.0**	*31.8*	41.9	41.4
NDSI238	**7.5**	33.9	54.1	*31.3*

**Table 5:** Estimates of effect sizes (partial eta squared) for source signal, bag and device ID on the recordings on the AudioMoth. Note that in multifactor ANOVA, as here, partial eta-squared values can sum to greater than one. Acoustic metrics as in Table 2.

	Effect Due to:		
Metric	Source Signal	Bag Used	Device and Its Filter
RMS	0.91	0.07	<0.01
dBZ	0.90	0.24	0.05
M	0.97	0.18	0.03
Bio	0.53	0.16	0.22
Smean	0.81	0.46	0.21
Ssd	0.04	0.02	0.05
Smedian	0.48	0.20	0.06
SQ25	0.36	0.19	0.04
SQ75	0.44	0.15	0.09
SFM	0.68	0.22	0.05
SH	0.80	0.22	0.05
TH	0.79	0.02	<0.01
Rough	0.79	<0.01	<0.01
Rugo	0.70	0.03	0.03
ADI	0.54	0.10	0.02
AEI	0.60	0.03	0.03
ACI	0.98	<0.01	<0.01
NDSI128	0.85	0.10	0.07
NDSI238	0.85	0.23	0.04

## Data Availability

The data are available from the University of Southampton data repository at DOI https://doi.org/10.5258/SOTON/D2399 (accessed on 19 August 2023).
